# Medical Uses of Wines

**Published:** 1858-12

**Authors:** 


*Medical Uses of Wines.* (From the British and Foreign Medico-Chirurgical Review.)

This is a subject thickly clouded with all sorts of prejudices and prepossessions, as is the discussion of most substances used equally by the sick and the healthy. Persons argue that what is good for themselves must be good for their patients. We have known a plethoric dietician, who himself loved lobster-salad and champagne in the small hours, advise a starveling dyspeptic to follow his custom of taking no breakfast till noon. So a hearty, rough-stomached doctor will declare one diluted alcohol just as good as another; the ascetic or the reformed rake will pronounce all equally bad; the gouty will dread all that is thin and acid; the aguish will have a predilection for Port.

It is very possible that prime wines may be made of all kinds, which may be equally and perfectly wholesome; but their rarity will always put them out of the reach of our patients, and what we have practically to think of in naming a wine for use, is at best a second or third rate article. We must also choose those which are capable of being grown in quantity proportioned to their popularity, or the chances of adulteration are exaggerated. When Madeira was on everybody's table, it could not be recommended to patients, for, in nine cases out of ten, it was either an inferior sort or a sour imitation. But now that it has gone out of fashion, a wholesome and often a very perfect wine is to be bought of that kind, and the adulterators expend their ingenuity upon Sherry. What we want is a liquor which is either produced in very large quantities, or is not sufficiently known to the million for it to be worth imitating.

The medical questions concerning the employment of wine will be put in the clearest light for exhibiting our real knowledge and ignorance, by considering separately the physiological effects on the human frame.

The effects may be practically included under the following heads: Exhilaration, Nutrition, Arrest of Destructive Metamorphosis, Inebriation, Degeneration of Tissue, Derangement of Digestion. The three first are good—the three last bad; and the object is to secure the former, while avoiding the latter.

Exhilaration is not merely a minor degree of drunkenness. It may be produced by many things besides alcohol, and which do not inebriate—such as, for example, the essential oils, peppermint, onions, valerian, assafœtida, tea, coffee. Even eating, and the increased circulation of blood, produce the effect to some extent. Alcoholic fluids truly do exhilarate with the greatest certainty and rapidity, but not in direct proportion to the alcohol they contain. A glass of wine will raise the spirits of a healthy person as much as a glass of gin; a glass of fine Claret as much as one of strong tavern Port; and this is not merely from the pleasure of taste or association, for the same may be observed in fever patients, whose gustatory nerves are blunted by a thick coating of sordes.

The distinction is not only a subjective one, evident to the mind of the recipient, while it is incapable of demonstration to others. There is a real physiological difference in the effects which follow exhilarating and intoxicating doses—a difference which, in its ultimate results, amounts to a complete contrast. The former increase the amount of vital powers rendered available in a given period, and the latter decrease them. Can there be a more perfect antithesis?

This is too important a matter to rest solely on the unassisted senses of patient or observer, and it does not do so, for the admirable experiments of Dr. Bocker have submitted it to the proof of chemical analysis. Though the whole series of his investigations into the action of alcoholic stimulants bear directly on the present subject, they are too mutually dependent on one another, and too lengthy for quotation. The general results, however, may be stated as follows:

1. The special action of alcoholic drinks is to arrest destructive assimilation—to stop the over active processes of life in the effects upon the organism; so that, for a certain period during the stay of the alcohol in the system, less urea, less phosphates, less water, are excreted by the kidneys, less carbonic acid by the lungs, and less digestion goes on in the alimentary canal, showing that the muscles, bones, nerves, &c., are not getting rid of their effete tissue, but retaining it, and making use of it as far as possible.

2. But, at the same time, they give rise in the body to a defensive reaction, which is prominent, first. immediately after taking the dose, then gives place to the special action, and on this ceasing, is again manifested to a greater extent.

3. So that if a suitable quantity be taken, and if both action and re-action are allowed to exhaust themselves before the dose be repeated, more manifestation of life, represented by more excretion and more consequent renewal of the body, takes place in a given time with the alcoholic drink than without. There has been a positive gain in vitality.

4. But if such a large quantity is taken at once that the re-action is overpowered, or if it is arrested by a continuous repetition of the dose, the manifestation of life is kept down; the body is not renewed because its effete particles are not removed, and the amount of vitality must certainly be reckoned at a loss.*

The first named state is Exhilaration, in which the alcohol may be fairly called a food or medicine, a medicinal food or dietetic medicine, for body and mind. The second state is Intoxication, when it is a poison to both.

Now, the exhilarating effects of diluted alcohol are very much increased by its admixture with sugar, extractive, vegetable essential oils, ethers, and the allied substances which have been described as producing the aroma and boquet of wines. With a quantity of alcohol which taken alone would be inefficient, a delicate wine is able to produce a decided impression upon the nervous system. When, then, this is mainly sought, as in cases of mental depression, hypochondriasis without bodily ailment, nervous exhaustion, over-anxiety, hysterical fainting, vomiting, and the like, or when wine is wanted merely to

*Beitrage zur Heilkunde, von F. W. Bocker, vol. i, sect. 6. Weingeist. We have introduced the name of this author again in our heading list, because he, and indeed all physiologists of the Schultz-Schultzenstein school, are much less known in England than they deserve. A collection of translations and abstracts would make an admirable volume for the Synenham Society.

smooth down the roughnesses of daily toil, we must remember that the good result may be obtained without the evil ; and we can obtain it with least chance of the evil by selecting liquors richest in their peculiar scented constituents. Bordeaux, Champagne, Rhine and Moselle wines offer a variety of choice, the first being the most perfect, and suitable to the greatest number of these cases ; whilst the others have certain inconveniences, hereafter to be mentioned, which often forbid their use in the special case to be prescribed for.

The beneficial effects on the nervous system are increased by effervescence ; thus, sparkling Champagne will sometimes have a most magical effect in stopping vomiting in cases accompanied with much nervous depression. And even in health, the greater exhilaration caused by genuine effervescing wines is notorious. The physiological explanation of this result is not very clear. It cannot be due to the carbonic acid alone, for the inhalation of this gas tends to completely opposite consequences. Perhaps the sudden physical change in the liquid during the extrication of the fixed air develops others which in a nascent state are more potent than at other times. Perhaps other gases are generated, whose properties are in themselves exhilarating. In the Champagne sent into Wurtemburg from Rheims, Baron Liebig found that for every volume of carbonic acid there were two volumes of protoxide of nitrogen* (laughing gas) ; and it was assumed, without absolute proof, to have been artificially introduced for the purpose of augmenting the joyous results of the bottle. The subject demands chemical investigation, on purely scientific grounds ; and it would, moreover, be useful to know, if we could thus at will increase the required exhilaration, while decreasing the quantity of alcohol or carbonic acid.

The gladening effects of alcohol are augmented by its mixture with other constituents of wine, but its intoxicating or poisoning effects are diminished. and thus more may be taken, with its advantages, and without its evils. So that, for example, if a man drinks a pint of Mr. Brande's Marsala, he gets a somewhat larger dose of spirit than there is in half a pint of gin,† but, it is unnecessary to say, without the same bad consequences. This is partly to be attributed to the pres-

---

* Medical Times, November, 1850.

---

†Marsala contains 26.03 per cent. of absolute alcohol (Brande); Geneva, 49.4 per cent. (Jones).

ence of the ethers* and sugar, but also in a great degree to the intimate combination of the alcohol with extractive and albuminous matter, so that it is not absorbed immediately by the membranes, but gradually and during a process of digestion. It is obvious that its local effect on the mucous surfaces and viscera must be thus much modified, and a powerful argument is afforded in favor of the use of wine instead of brandy for invalids.

Nutrition is an indirect effect of wine. There is shown by chemical investigation to be very little substance in it capable of building up the body. The phosphates and albumen are more readily found elsewhere, as Franklin has imprinted on our memories by his comparison of a penny roll and a gallon of beer. But alcohol seems to render the alimentary canal more ready to absorb nutriment. Farmers find this, and always try to put some waste beer or fermenting grains in their pig troughs. Physicians find it, too, and give their patients cod-liver oil in a glass of Sherry when they would have them fatten quickly. The effect, however, is probably confined to oleaginous food and the adipose tissue, for the digestion of albuminous matter by the gastric juice is certainly impeded by alcohol.

Hence, we gain the following rules concerning the administration of wine as an aid to nutrition :

1st. That the alcoholic contents are those of principal importance, and that the amount of solid or nutritive matter in the wines makes but little difference.

2dly. That we may hope help from it in increasing adipose tissue, but not muscle.

3dly. That, as its agreement with fatty food is the prime object, we must avoid those wines which are likely to make such food unassimilable, as, for example, by making it rancid.

And, therefore, 4thly. That sound wines with a small proportion of acid to their alcohol, and but little body to cause re-fermentation, should be selected ; the types of perfection may be considered the dry Spanish wines, Amontillado and Manzanilla.

And, 5thly. They should be taken along with the fatty food itself, or immediately after it.

---

*The disinebriating influence of ether is shown by its being actually a remedy for drunkenness. Twenty or thirty drops taken neat on a little oil will restore to temporary sobriety. The knowledge of this fact has been popularized in France, by its forming a point in a wicked railway novel (Le Trou de l'Enfer), the author of which perhaps owed it to M. Batilliat (Traite sur les Vins de la France, p. 190).

The arrest of destructive metamorphosis, or what has been picturesquely called "the moulting of the tissues," is unquestionably the most important of the medical uses of alcoholic liquids. By them we are enabled to stay the progress of interstitial death in low fevers, till the period of the zymotic poison's virulence is passed, and it has either been evacuated or become inert. By them we can check the exhaustion of the body through excessive secretion, as in cases of chronic catarrh, ulcers, abscesses, amputations, &c. By them we can diminish in ordinary dietetics, the wearing out of the body by the over-worked mind, which, in this busy metropolis, throws so many into the hands of the physician. But in the wielding of this two-edged sword, the greatest judgment is requisite, lest we carry the effect too far. The destruction of effete tissues is part of life, and necessarily precedes constructive renewal; if, then, we check it too far, interstitial life is diminished, and the system is overloaded with matter incapable of vitality.

It is better, therefore, to give alcohol in a diluted form, even when we wish to produce its most decided action, as in typhus fever, for example. And it is better to give it combined, as it is in wine, with other substances of partially corresponding action, than to administer it merely diffused in water, as is sometimes done for economy's sake. Sugar, we know from Dr. Bocker's experiments, has a special effect in limiting the destruction of tissues containing phosphates, tissues of no less importance than the bones and nerves. And it is likely that similar investigations into the physiology of ethers may show some special effects belonging to them. The acids, too, and the extractive in wines, seem to prevent, better than water, those injurious effects upon the mucous membranes which spirituous liquors exhibit. There is, then, no extravagance in preferring wine to brandy and water in the management of low fevers in hospital and parish practice.

This is not the place to discuss details in the mode and period of administering wine in acute complaints. But one rule may be deduced from the view taken of its physiological action, viz: to allow intervals to elapse, during which its effects may subside, and the system recover for a time its metamorphoses, so that the effete tissues may have a due exit. The night is a convenient time for this in general; but if, from any cause, that is considered expedient, some hours of corresponding duration should be selected, during which the administration of stimulants may be discontinued.

The wine chosen for fever cases is usually Port; but the rarity of really good Portugal wine, and the excessive badness of all low-priced imitations now in the market, render it daily more and more incumbent upon us to have substitutes at hand. The best in the London market seem to be the red Spanish wines, Beni Carlo and Cadiz; especially the former, which, indeed, is often mixed with spoiled Portuguese wine, and sold as Port. It may be had at a low price, considering its strength, and is highly to be commended for hospital use in a diluted state.

Poor people, however, are not the only patients supplied with Port wine unfitted for the sick room. The prepossession in favor of antiquity causes many cellars in wealthy houses to furnish nothing but a damaged article. To find fault with a bottle that cost a great sum a great many years ago, is flat heresy; and the better way is to give it up at once, and order your patient a good full bodied wine of a different nature, such as Madeira, Burgundy or Hermitage.

Inebriation is a terrible word to meet with in periodical literature. It opens up a prospect of so many social and political questions, that the reader is apt to close the page in despair. He shall be let off here with a simple remark derived from wayside observation—viz: that in all countries where wine is plentiful and cheap, drunkenness is almost unknown; where it is most expensive, that vice is at its maximum.

Degeneration of tissue, as a consequence of drinking, appears to be a chronic state of that arrest of metamorphosis which has been already discussed as a remedy for disease. The effete tissue remains as a useless burden mixed up with the healthy, and is finally converted into the least vitalized of all the organic constituents of the body, oil or fat. Careful and valuable observations have been made by Dr. Bocker, on the abnormally retained blood-discs in the circulating fluids of habitual spirit drinkers, and the appearance of the degenerated hearts, livers and kidneys of these miserable suicides is familiar to us all.

Degeneration arises from the arrest of metamorphosis being too long and continuously kept up. Hence, there is little danger of it in acute cases, where the large quantity of alcoholic remedies we find it expedient to administer is necessarily diminished as the disease recedes, and during convalescence is reduced to the ordinary allowance of health. But in chronic cases it is often a matter for serious consideration whether we shall employ an agent capable of doing, along with the good we intend, an evil greater than that originally to be combatted. If the dose of a stimulant be repeated before the arrest of metamorphosis has ceased and the reaction of the system has begun, a second arrest indeed takes place as before; but the postponed reaction is augmented in force each time it is delayed, and when it occurs at last, it is so painfully depressing that it becomes more and more difficult to resist the instinct to put it off, and in the end it is really dangerous to do so suddenly. This is the short history of confirmed tippling; and often, we fear, it may be traced in its origin to the carelessly worded advice of some medical man. Science or practice has taught him that alcoholic action will alleviate certain morbid phenomena, and he recommends it without due warning. The patient knows no harm in alcohol except drunkenness, and so long as he avoids that vice, thinks he cannot keep up too steadily the agreeable relief he experiences.

Alas! much safer for him would be the occasional debauch of a man he despises as a profligate, than his own continuous steady course towards death. A drunken bout brings its own cure, and is usually allowed to be followed by reaction afterwards; but the most alarming symptom in a tippler is, that he cannot get drunk. Day by day there is a little less life in his system, till at last his degenerated body is fit for burial.

Now, the results above described are, practically speaking, unknown as the consequence of wine; it is the spirit drinking that leads to them. There are several reasons for this, independent of the chemical differences of the liquors. Wine is rarely used except at the principal meal, or as a sort of medicine in measured quantity at other hours, so that the effects have time to pass away before another dose becomes due, and no craving for increased quantity is experienced. In fact, men go on taking daily for quarters of their life the same identical number of glasses, feeling daily the same comfort, and never finding it necessary to increase the quantity. But the spirit bottle is opened when its owner "feels to want it,"—nay, it is very often carried about the person under the appropriate name, as regards its deadly results, of a "pocket pistol."

We have been in the habit, in insurance practice, of omitting the usual enquiries about "sobriety" and "temperance," &c., which give offence and elicit no information, and substituting for them the simple question—"Do you ever take spirits between meals?" This is something definite, not to be shirked, and if answered in the affirmative, should lead to rejection.

The subject of spirit drinking takes up more space in this article than our promise of avoiding temperance common places perhaps led the reader to expect. But we have two excuses: one is, that it occupies quite distinct ground from the question of drunkenness, has much more to do with the production of disease, and is, therefore, much more the province of a medical reviewer. The other excuse is (we blush to write it,) that no class of persons who have received a liberal education are so often addicted to it as medical men Londoners were shocked two or three years ago at the suicide of a highly moral and intellectual surgeon, who left a paper attributing his despair to the habit of secret tippling; but they would have been less astonished had they known how many practitioners all over the country suffer from the peculiar dyspepsia of alcoholism. The long robe and her majesty's uniforms are occasionally disgraced by inebriation, clergymen may sit too long at the bottle, but spirit tippling seems left to medical men and the classes below them. They have many temptations; hard mental and corporeal toil, sudden calls for exertion when tired, broken rest, irregular exposure to cold and wet, weary waiting in lone farm-houses for lingering labors, the dull company of ill-educated persons, the wish to be sociable and not seem proud, are a few of them. Into these temptations they do fall, and that on a large scale, especially in the rural districts.

To require of an unfortunate patient and brother practitioner that he should give up at a blow that alcohol, which instinct and science agree in teaching him to be necessary, is too great a demand. If he became a teetotaller, he would probably die all the sooner. Hard common places about the virtue of temperance and the evil of its opposite, produce no more effect than school boy's themes. What he wants is—first, kind sympathy with his misfortune, and second, a rational means of getting rid of it. Now, nothing contributes more towards the latter than a clear sketch of the chemistry and physiology of the subject, and a belief that the advantages of alcohol may be had without its disadvantages. He should reflect how wine differs from the spirits which are in it; and again, how it is not so much the quantity but the frequency of the dose, which is hurrying him to the grave, and his children to poverty. The most complete relief is the substitution of wine for spirits. The very economy which was perhaps the first origin of the habit, will prevent excess in the dearer liquid. If that cannot be accomplished, let at all events drams between meals be avoided as poison; and let the addition of sugar, and flavors in the shape of lemon, fruit, or a few drops of nitric ether, make the drink approach a step nearer to the juice of the grape, and be daily more and more diluted.

Among the derangements of digestion arising from wine, it will not be necessary to dwell long upon the immediate consequences of a debauch. It is usual, in army medical returns, to report it as "febris," as indeed there is, truly enough, an ephemeral fever; but, like other fevers, it works its own cure, and civilians are not in the habit of applying to it the same euphonistic nomenclature. But, without being taken in such quantity as to be considered an excess as regards alcohol, wines will sometimes cause a disturbance of digestion, which prevents our sanctioning their use in cases where otherwise we might be willing or anxious to do so. This is always accompanied by the presence of a large quantity of acid in the alimentary canal.

In some instances this excessive production of acid follows equally all sorts of wines, and even spirits. Then it is due to the mucous membrane of the stomach being so morbidly sensitive that it becomes irritable and temporarily inflamed, so that it refuses to secrete its solvent juice, and to perform with sufficient activity the peristaltic movements. Hence, the alimentary mass undergoes the acetous and lactic fermentatious, instead of being digested. These patients ought to abstain from all alcoholic drinks whatsoever, till cured of their morbid condition.

More commonly it follows only wines, and some sorts of wines more than others. These cases deserve much thought, because they are in danger of falling into the snares of spirit drinking, and also, because very often the patient's system specially requires a stimulus which yet he cannot take without inconvenience. When we reflect on the large quantity of free acid existing in wine, cannot be surprised at its causing some trouble in the stomach. If a man drinks half a bottle of Hock, he swallows one hundred grains of acid, equal to five tablespoonfuls of lemon juice; in a pint of Claret, eighty grains; in sparkling Champagne or Madeira, the same amount; in Port, if he takes even this comparatively large allowance, he does not get above sixty grains; but then in the three last there is nearly an ounce of sugar, which, mixed up with the food, has a strong tendency to ferment, and turn into a fresh portion of acid at a more advanced period of digestion.

Here chemistry steps in with a valuable aid. In the simple instrument of a standard solution of caustic soda, we possess a means of testing rapidly the whole acid contents of wines, and rejecting any which are thus declared unfit for our patient.

But it makes some difference what sort of acid is contained in the wine. Acetic is to many stomachs much less injurious than tartaric, and it is found that the proportion of these to one another varies very much in the products of fermentation. Thus, in Madeira nearly one-third of the acid contained is acetic; in Port, only one-fourth; in Claret, one-fifth; in Champagne, one-seventh; and in Hock, not one-eighth, whilst the rest is the least digestible, tartaric, or its ally, racemic.* Besides these, the tannic must be allowed for, small indeed in quantity, but powerful in operation, as its use in medicine shows.

Of course, both the quantity of acid and the proportions of the several acids vary, within certain limits, in different specimens even from the same vineyard, and still more in growths classed undeɹ a common name in the market. So that to give an opinion as to the fitness of a particular wine for drinking, we must carry our investigation rather farther than merely the application of the soda test.

The acetic acid may be estimated by distilling it off from the wine slowly, at a moderate temperature, so as not to decompose the extractive, and measuring it by the standard alkaline solution.

Sugar in wine, which is to be taken by itself as a medicine, is beneficial by making the acid and alcohol less immediately irritating to the mucous membrane; but in that which is to be mixed with food it is very apt to increase the generation of acid in the stomach or cæcum to an injurious extent, generally two or three hours after meals. If an examiner of wine is disposed to reckon the absolute quantity of sugar, he will have to go to the expense of Soliel's saccharometer (which costs, with its accessories, not much under £20,) and even then may have his analysis doubted by a chemist;† but a fair comparative valuation may be made by first neutralizing the acids with lime, and estimating the sweetness which remains by the taste. This is done by measuring the quantity of water which requires to be added before all traces of it cease to be perceptible to the palate.

---

*See Mulder, p. 202. In 100 grammes of wine there were—

| | Milligrammes of acetic acid. | Milligrammes of tartaric, racemic, &c. |
|---|---|---|
| Madeira, | 167 | 310 |
| Rhine Wine, | 66 | 480 |
| Port, | 95 | 283 |
| Bordeaux ordinaire, | 86 | 390 |
| Champagne, | 64 | 409 |

†The fallacy in Soliel's polarizing saccharometer as a quantitative test is, that uncrystallizable sugar rotates the ray to the left, whilst glucose and cane-sugar rotate it to the right. So that a sample of Sherry, for example, with its usual allowance of the uncrystallizable, might be so adulterated with white lump molasses, caramel, or malt, as exactly to balance, and appear to contain no sugar at all.

The injurious effect of ill-prepared effervescing wines is easily explained by the large quantity of undecomposed ferment they contain. This is set in action by the warmth of the alimentary canal, and can hardly be overcome even by the strongest digestive powers. Flatus and acidity are its normal consequences.

The proverbial unwholesomeness of "mixing wines" is not explained by chemistry. In most cases the evil may be traced to the temptation to increased quantity, or to the taking of some sorts which, even if adhered to throughout the meal, would be equally hurtful. In fact, the precept of keeping to one wine seems to rest on the same principle as keeping to one meat.—*Brit. & For. Medico-Chirurg. Review.*

